# *There’s an App for it*: A systematic review of mobile apps providing information about abortion using a revised MARS scale

**DOI:** 10.1371/journal.pdig.0000277

**Published:** 2023-07-17

**Authors:** Bianca M. Stifani, Melanie Peters, Katherine French, Roopan K. Gill

**Affiliations:** 1 Department of Obstetrics and Gynecology, New York Medical College, Valhalla, New York, United States of America; 2 Vitala Global Foundation, Vancouver, British Columbia, Canada; 3 Department of Obstetrics & Gynecology, University of Toronto, Toronto, Ontario, Canada; Iran University of Medical Sciences, IRAN (ISLAMIC REPUBLIC OF)

## Abstract

Mobile applications (apps) are increasingly being used to access health-related information, but it may be challenging for consumers to identify accurate and reliable platforms. We conducted a systematic review of applications that provide information about abortion. We searched the iTunes and Google Play stores and queried professional networks to identify relevant apps. To evaluate the apps, we used the validated Mobile App Rating Scale (MARS) and added relevant abortion-specific elements. Two reviewers independently rated each app, and we report mean scores on a 5-point scale across the domains of engagement, functionality, esthetics, and information. We also rated app characteristics (including target population and reach), and number of desirable abortion-specific features. We defined recommended apps as those that achieved a score of 4.0 or above for the question: “would you recommend this app to people who may benefit from it?” Our search initially yielded 282 apps and we identified two additional apps through professional mailing lists. Most were irrelevant or not abortion-specific. We excluded 37 apps that sought to discourage users from seeking abortion. Only 10 apps met inclusion criteria for this review. The Euki app had the highest overall score (4.0). Half of the apps achieved a score of 3.0 or greater. Most of the apps had few desirable design features. Some apps provided significant information but had poor functionality. Only four apps met criteria for being recommended: Euki, Safe Abortion by Hesperian, Ipas Mexico, and Marie Stopes Mexico. In conclusion, we found few apps that provide unbiased information about abortion, and their quality varied greatly. App developers and abortion experts should consider designing additional apps that are clinically accurate, unbiased and well-functioning. We registered this review in the PROSPERO database (Registration # CRD42020195802).

## Author summary

Mobile applications (apps) are a popular means to obtain information about health issues. However, not all apps provide accurate information to users. Here, a team of specialized doctors and app developers searched for apps that provide information about abortion and scored them using an adapted version of an existing scale called the Mobile App Rating Scale (MARS). While we initially found 282 apps, we excluded most because they were irrelevant or not specific to abortion. We excluded 37 because they were designed to discourage users from having abortions. We only included 10 apps in this review and found that only four could be recommended. The Euki app had the highest overall score. Few apps had desirable design features and several apps provided information but did not work well or were poorly designed. We conclude that clinical experts and app developers should consider collaborating to design apps that are accurate, well designed, and functional.

## 1. Introduction

As global smart phone use expands, mobile applications (apps) are increasingly being used to access health-related information and even healthcare services. Apps provide an added benefit over other sources of information as they often include interactive features and customizable components. During the COVID-19 pandemic, the use of medical apps increased significantly [1], while healthcare visits for preventative services decreased [2].

Given the proliferation of health-related mobile apps, it is sometimes challenging for consumers to identify accurate and reliable platforms to serve their needs. Similarly, it may be challenging for app developers to know where the remaining gaps are in terms of developing new platforms. This has prompted some researchers to attempt systematic analyses of existing apps covering various health topics. For example, researchers have reviewed apps for weight management [3], self-management of diabetes [4], and mental health [5].

One group of researchers developed and validated a generic scale to assess mobile applications developed for any health topic (the Mobile App Rating Scale, or MARS scale) [6]. Other researchers have since applied it to several types of health-related apps, including apps for checking drug-drug interactions [7], apps for parents of infants admitted to neonatal intensive-care units [8], and mindfulness apps [9].

In the field of reproductive health, mobile apps have been developed that focus on a variety of topics, from fertility and pregnancy tracking to contraception and sexually transmitted infections [10]. Reviews of reproductive health apps have so far focused on apps for pregnancy prevention [11], and for adolescent pregnancy prevention, more specifically [12]. One study undertook an evaluation of a single app, the Teens in NYC mobile phone app, which provides information on a number of sexual health services, including abortion [13]. Another reviewed family planning apps in general, but was limited to English language apps [10].

Abortion is often considered to be a sensitive and controversial topic. Obtaining accurate information about it can be challenging, particularly since access is legally restricted in many contexts. Limited data exist about how and where individuals seek information about abortion, and whether the sources they utilize include mobile applications among other forms of digital media. The authors of a scoping review of mobile apps for family planning published in 2019 found that although mobile apps for abortion existed, there was no research on this topic [10]. The objective of this review is to describe the quality, quantity, and geographic focus of existing apps that provide information and aim to increase access to abortion. By mapping what is currently available in the mobile app market to persons seeking abortion, we also aim to identify remaining gaps that can be filled by designing new apps.

## 2. Methods

**Fig 1:**
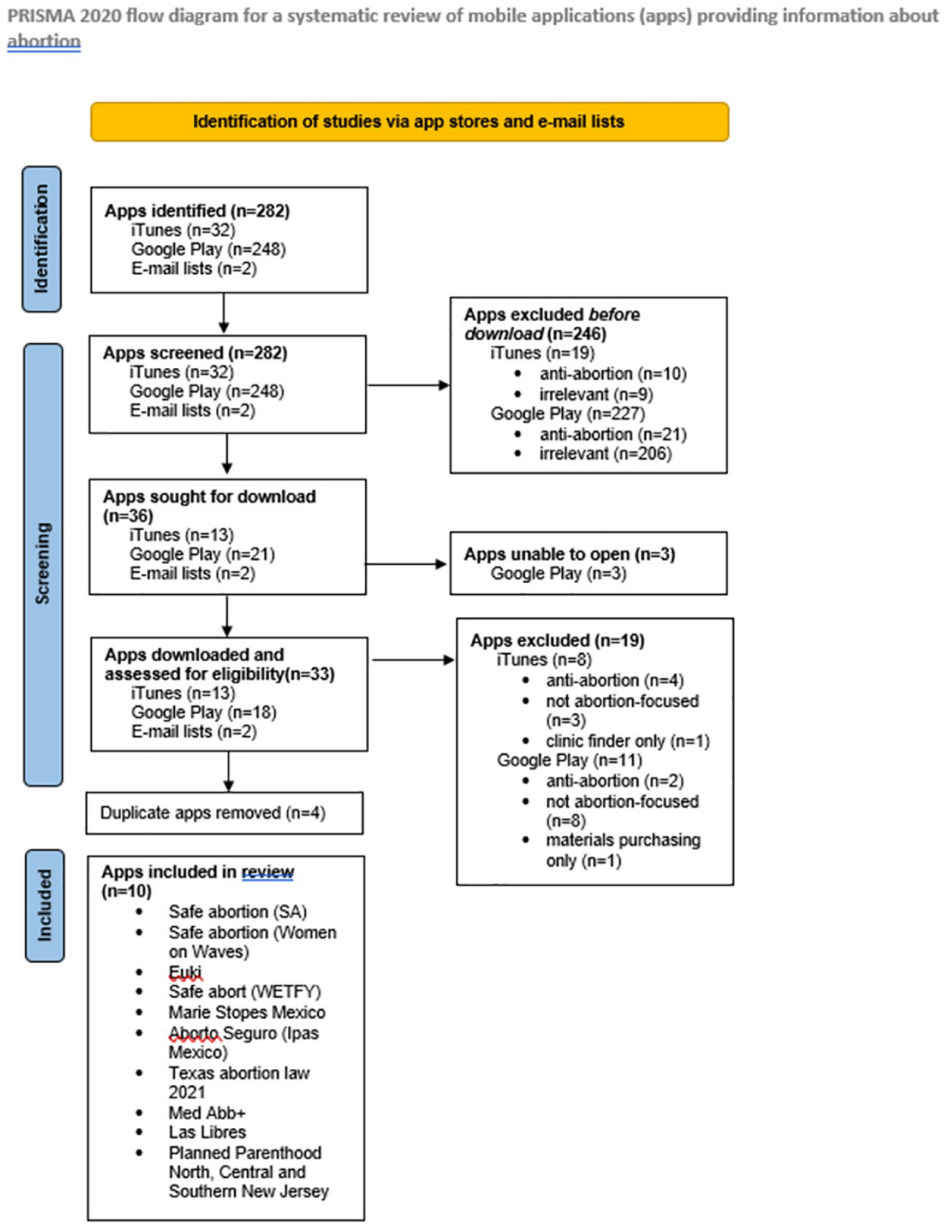
PRISMA Flowchart.

Although an app review differs from a classic systematic review in terms of search strategies and content evaluation, we followed the PRISMA guidelines (see Fig 1) for reporting of systematic reviews as much as possible [14]. Prior to starting the review, we registered our protocol in the PROSPERO database (Registration # CRD42020195802).

### Eligibility criteria

For the purposes of this review, eligible apps were those that had a primary focus on abortion, which we defined as at least 25% of the app content being related to abortion. We included apps that provide information about abortion or aim to increase access to abortion. We intentionally excluded apps developed by abortion opponents including pregnancy crisis centers and others whose intention appeared to be to discourage people from seeking abortion. We define these apps as “anti-abortion apps” in the flow chart of abortion app results. We also excluded apps that were abortion clinic finders only and did not provide information about abortion.

### Information sources and search strategies

Using smartphones and tablets, we searched the Google Play and Apple iTunes stores for abortion-related apps. We conducted the original search in July of 2020 and updated it in October of 2021. We selected these two sources as these sources cover a great majority of the mobile app market. To ensure that we were not missing apps based on individual researchers’ locations, we turned off our device location settings and conducted parallel searches in the United States, Canada, the Dominican Republic, and Germany. We also e-mailed two international listservs of abortion experts to ask about apps related to abortion. By doing so we decreased the risk of missing relevant apps based on the search terms we selected, and we included apps that were not listed in the app stores.

We tested several search terms. We initially included broader terms such as “family planning,” but later decided to exclude them because the Google Play and iTunes store search features have low specificity and the results yielded too many irrelevant apps. Because we were looking for apps specifically focused on abortion, we selected the terms “abortion” and “pregnancy termination.” We initially searched for these terms in six languages (English, Spanish, Portuguese, French, German, Italian) that our team is fluent in, but noticed that searches in languages other than English did not yield any additional relevant apps. We also saw that the term “pregnancy termination” did not yield any additional relevant apps. We therefore only selected the term “abortion” for the final search.

### Selection process

**Fig 2:**
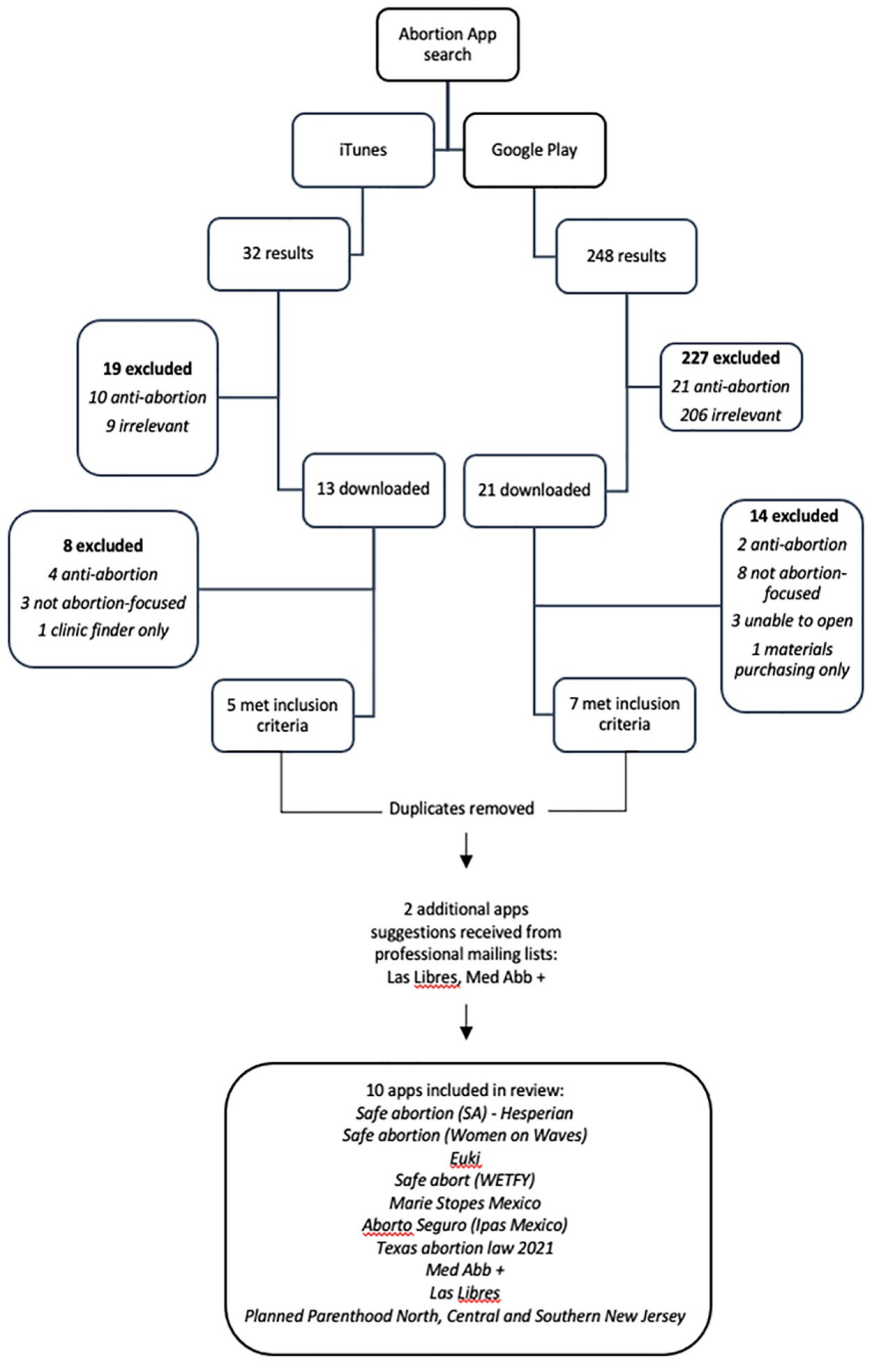
Flowchart of Abortion App Search.

Two researchers screened all apps based on the app description and downloaded apps that appeared to meet the inclusion criteria. We later excluded any downloaded apps that did not meet the inclusion criteria (see Fig 2).

### Assessment of applications

To rate the quality of the apps, we used the MARS scale [6]. This scale was developed to evaluate the quality of mobile health apps. It evaluates apps in four main domains: engagement, functionality, esthetics, and information. It also includes a subjective quality domain and an app-specific section, which allows for adjustments based on the type of app that is being reviewed. Researchers have tested the scale and shown that it has excellent internal consistency and interrater reliability [6].

We rated abortion apps using the MARS scale, to which we added sections to evaluate apps’ global reach and the quality and quantity of abortion-related content. First, we added a “characteristics” score, which aims to measure each app’s reach, and includes factors such as number of languages and user reviews (see Box 1). Second, we added abortion-specific information to the information section because, as clinical specialists in abortion care, we wanted to measure how many important abortion-related topics were covered in each app (see Box 2). Third, we added a “desirable features” section in which we assigned points based on the number of interactive features present in the apps (see Box 3).

**Box 1. Scoring rubric for app characteristics**

1. Platform (1 point if iOS only or Android only, 2 points for both)
2. Target audience (1 point for local, 2 points for regional, 3 points for global)
3. Languages (1 point for one language, 2 points for two, 3 points for more than two)
4. Average rating (1 point for three stars or less, 2 points for four stars, 3 points for five stars)
5. Number of ratings (1 point for less than ten, 2 points for ten to twenty, 3 points for more than twenty)
6. Number of downloads (1 point for less than 1000, 2 points for 1000–4999, 3 points for 5000–9999, 4 points for more than 10,000)

**Box 2. Scoring rubric for abortion-specific information**

***General* abortion information specific score: Does the app cover each of the following elements (1 point each)**

*Pre*-*abortion*

- Counseling, decision-making
- Eligibility

*Abortion*

- Medical abortion: mifepristone, misoprostol
- Surgical abortion
- Pain control

*Post*-*abortion*

- What to expect (normal)
- Complications / warning signs
- Contraception
- Follow-up
- Emotional / well-being

General abortion information specific score: ____ / 10

***Useful* abortion information specific score—Are the following present in the app? (1 point each)**

- Location-specific information on abortion laws?
- Abortion cost information?
- Does the application send abortions pills to the individual?
- Is there information about where to get an abortion?
- Is there information about how to avoid fake clinics?
- Is there information about how to avoid fake pills?
- Is there information about self-managed abortion?
  - If yes, is there information about self-assessing for eligibility?
  - Is there information about where to get abortion pills?
  - Is there information about how to use abortion pills?
  - Is there information about self-assessing for success of abortion?

Useful abortion information specific score: ____ / 10

**Box 3. List of desirable app features**

- Privacy / Security–is there an option to register and create a password?
- Direct communication–is there a chat feature for users to directly communicate with counselors or providers and ask specific questions?
- Geo-localization–is there an option to enter one’s location and search for nearby resources?
- Gestational age calculator–is there a feature that allows users to enter their last menstrual period and that calculates their gestational age?
- Frequently asked questions–is there a list of frequently asked user questions somewhere within the app?
- Are there user testimonials somewhere within the app?
- Is there a symptom diary–ie, a part of the app that allows users to track their physical and/or emotional symptoms?
- Is there a search feature?
- Is there a bookmark feature?
- Is there an option to delete data stored in the app?
- Is there a reminders feature?
- Are there links to emotional health resources?

We used these new sections instead of the app-specific section of the original MARS scale, which we found to be less useful for evaluating abortion apps due its focus on behavior change. We made these additions to the scale based on our team’s expertise. BMS and RKG are obstetrician-gynecologists with expertise in abortion care. RKG also has experience in the development of digital content. In partnership with UX/UI designers, she developed a web-based tool for post-abortion care in Canada, and a mobile app to support self-managed abortion in Venezuela. From her work engaging stakeholders to co-design an abortion app in Venezuela, we learned that users desire specific features from digital tools. For example, security was a key concern for prospective users in Venezuela, as were emotional health resources. These findings helped guide the “list of desirable app features” section of our revised MARS scale. For this review, the whole research team met and agreed on the revised MARS scale criteria prior to initiating the app reviews.

Each item on the MARS scale is scored from 1–5. Items we added to the scale did not necessarily score as 1 through 5, but we later converted the scores for each category as 1 to 5 to facilitate comparisons across domains.

### Data collection and synthesis methods

Two reviewers independently rated each app using the revised-MARS scale. We then combined all the ratings into a single Excel spreadsheet which we used to calculate average scores per section, which is what we report in this manuscript. Where total average scores for an app differed by more than ten points on the raw scale (which had a total of 137 points), a third reviewer reviewed the app, and we report average scores calculated using all three reviews. The team of app reviewers included two clinical abortion specialists (BMS, RKG), of which one is also an app developer (RKG), and two obstetrician-gynecologist trainees (MP, KF).

## 3. Results

We found a total of 32 apps through the iTunes store, and 248 apps through the Google Play store. Of these, 215 were irrelevant and 32 were explicitly anti-abortion. We did not exclude any apps based on language, because all the apps we found were in either English or Spanish (some had additional languages but included English and Spanish). We downloaded 34 apps and excluded an additional 22 that were either anti-abortion, not abortion-focused (less than 25% of the content related to abortion), or clinic finders only. After removing duplicates, a total of eight apps from our search met inclusion criteria for this review, and we received two additional app suggestions from professional mailing lists. We therefore reviewed 10 apps providing information about abortion (See Fig 2).

More than two thirds of the apps had a specific geographic target area (7/10), while the remainder had a broader audience and could be customized in terms of location and/or language. Most of the apps targeted lay-persons, and only one app (Aborto Seguro) specifically targeted healthcare providers. Several of the apps had very few or no ratings. Most of the apps had been downloaded relatively few times on Google Play (the only platform that provides this information), with Safe Abortion by Hesperian being the only app that had more than 100,000 downloads. See Table 1 for app characteristics.

**Table 1:** Characteristics of apps included in a systematic review of apps providing information about abortion.

App name	Developer	Platform	Language(s)	Target audience	Average rating^1^	Downloads^2^
Safe Abortion (SA)	Hesperian Health Guides	Android, iTunes	English, Spanish, French, Igbo, Kiswahili, Yoruba, Luganda, Kinyarwanda, Amharic, Oromo	Laypersons, healthcare workers, women’s health advocates in multiple countries	3.1	100,000+
Safe Abortion with pills	Women on Waves	Android, iTunes	Afrikaans, Indonesian, German, English, French, Xhosa, Italian, Hungarian, Malay, Dutch, Polish, Portuguese, Russian, Sinhala, Swedish, Tagalog, Turkish, Tamil, Arabic, Farsi, Chinese, Korean	Laypersons, healthcare workers, advocates in multiple countries	4.6	10,000+
Euki	Women Help Women	Android, iTunes	English, Spanish	Laypersons in the United States	4.8	1000+
Safe Abort	WETFY / PSI India	Android	Hindi, English, Oriya	Laypersons who are users of a mifepristone-misoprostol combination pack available in India	None	10,000+
Marie Stopes***	Fundación Marie Stopes México	Android, iTunes	Spanish	Laypersons in Mexico	None	100+
Aborto Seguro	Ipas México	Android, iTunes	Spanish	Healthcare professionals in Mexico	5.0*	10,000+
Texas Abortion Law 2021	Senussi 69	Android	English	Unclear	None	10+
Med Abb+**	Familienplanungszentrum Berlin e.V.	iTunes	German, English, Spanish, Italian, Polish, French	Laypersons who receive medication abortion from a clinic in Berlin, Germany	None	Not available
Libres**	Las Libres	Android	Spanish	Laypersons in Mexico	Not available	Not available
PPNCSNJ***	Momedex, Inc	iTunes, Android	English	Laypersons in New Jersey, USA	1.0*	1000+

^1^For apps that had ratings on Google Play and iTunes, we present the average rating across the two platforms. Some apps did not have any ratings.

^2^ Number of downloads information was only available on Google Play

*Based only on one review / rating

**Apps found through professional listservs not through app store

***Apps that link to website and do not function offline

Table 2 shows mean revised-MARS scores across each domain and overall. Three reviewers reviewed Marie Stopes Mexico, PPNCSNJ, Safe Abort, and Texas Abortion Law, while two reviewers reviewed the remaining apps. The Euki app had the highest overall score (4.0), while the Texas Abortion Law app had the lowest (1.4). Safe Abortion by Hesperian scored relatively high on information (3.5), but low on engagement (2.7). Similarly, Safe Abortion with pills scored relatively high on information (3.3), but low on functionality (2.9). Most of the apps had few, if any desirable features and therefore scored low in this category. Only the Euki app had more than 3 desirable features and scored high in this category (4.2). The most common interactive features we encountered were gestational age calculators and “frequently asked questions” sections. Of note, two of the apps (Marie Stopes Mexico and PPNCSNJ) linked to the organizations’ websites and did not function offline. The scores therefore reflect the websites’ characteristics. The Safe Abort app by WETFY was unique in that it was activated by pointing the device camera to the SafeAbort kit symbol, and then it played a video, but it did not function as a typical app.

**Table 2:** Mean revised-MARS scores of reviewed abortion apps.

	Characteri-stics	Engage-ment	Functiona-lity	Esthe-tics	Informa-tion	App Subjective Quality	Desirable Features	Total points
Euki	3.6	4.3	4.4	4.0	3.6	4.3	4.2	4.0
Safe Abortion (SA)	4.6	2.7	4.5	3.8	3.5	3.8	1.7	3.4
Aborto Seguro	4.2	3.8	4.8	4.2	3.2	3.9	0.4	3.3
Safe Abortion with pills	4.7	2.7	2.9	2.3	3.4	3.3	1.3	3.0
Marie Stopes Mexico*	1.6	3.2	3.2	3.6	3.5	3.4	1.9	3.0
PPNCSNJ*	2.2	2.8	3.8	3.1	3.5	2.8	1.5	2.9
Safe Abort*	3.9	2.7	4.2	4	2.9	2.4	0	2.8
Las Libres	0.8	3.0	3.8	2.3	3.1	3.1	1.7	2.7
Med Abb +	1.9	2.7	3.0	3.3	2.4	2.1	1.7	2.4
Texas Abortion Law*	1.4	1.9	3.3	2.0	1.1	1.1	0	1.4

*Apps reviewed by 3 reviewers

Table 3 highlights our reviewers’ average scores to the question “would you recommend this app to people who might benefit from it?”. Only four apps had an average score across all reviewers that was 4.0 or higher and, therefore, met our criteria for being recommended. The Euki app had the highest score in this category and therefore received the strongest recommendation from our reviewers.

**Table 3:** App Subjective Quality scores.

	Would you recommend this app to people who might benefit from it?	
	Average score	Corresponding response
Euki	4.5	There are many people I would recommend this app to
Safe Abortion (SA) (Hesperian)	4.0	There are many people I would recommend this app to
Ipas Mexico	4.0	There are many people I would recommend this app to
Safe Abortion (women on Waves)	3.5	Maybe; there are several people whom I would recommend it to
Marie Stopes Mexico*	4.0	There are many people I would recommend this app to
PPNCSNJ*	3.3	Maybe; there are several people whom I would recommend it to
Safe Abort*	2.7	There are very few people I would recommend this app to
Las Libres	3.5	Maybe; there are several people whom I would recommend it to
Med Abb +	2	There are very few people I would recommend this app to
Texas Abortion Law*	1	Not at all; I would not recommend this app to anyone

## 4. Discussion

In this review, we found relatively few apps that provide information about abortion. The quality of these apps, measured using a revised version of the validated MARS scale, varied greatly, and our team of reviewers recommended only four of the ten apps. This information is new to the scientific literature because this is the first systematic review of abortion-related mobile apps and, to our knowledge, the first research paper to specifically examine abortion-related mobile apps.

An important finding of this review is that several apps contained accurate information yet had low functionality or esthetics scores. This underscores the importance of developing apps in partnerships with clinical abortion experts and design professionals, as poor app functionality and esthetics are likely to decrease an app’s potential impact. Also, some of the apps are not available offline and link to existing websites instead, thus adding little value than what is already provided by an organization’s website. Our team also evaluated whether apps contained helpful, abortion-specific interactive features, such as gestational age calculators or reminders to take abortion pills. We found that only one app had more than three desirable features, while most had none to one feature. Mobile apps are interesting digital tools because they are more interactive than web-based content. Yet most currently available abortion apps are underutilizing interactive features, likely limiting their appeal over that of traditional web-based content.

An unexpected finding of this review is that our searches of the Google Play and iTunes stores yielded more than three times as many anti-abortion apps as apps that provide information about and facilitate access to abortion. This is new to the literature as no studies have examined the prevalence of apps that discourage individuals from seeking abortion. However, our results echo what other authors discovered while searching the Internet for abortion-related information. One group reviewed the contents of the top websites that resulted for a Google search for “abortion pill.” They found that three of the four top webpages were anti-abortion and that these pages provided a variety of disinformation about the abortion pill [15]. Another group performed Internet searches for abortion services in the 25 most populous US cities, and found that 13% of webpage results, 22% of maps, and 30% of ads hindered abortion self-referral [16]. Previous studies have also shown that misinformation about abortion abounds on the Internet. Crisis pregnancy center websites provide false information, most commonly about the link between abortion and mental health, preterm birth, breast cancer, and infertility [17]. Many do not explicitly state that they do not provide abortion [18].

It is concerning that our research team downloaded several anti-abortion apps and only discovered upon close inspection that they sought to discourage persons from seeking abortion. Laypersons who are seeking information about abortion may be unable to distinguish apps that provide unbiased information from those that were specifically designed to discourage them from seeking abortion. Future research should examine to what extent consumers can discern the quality and intent of the sources they consult.

This study has several limitations. First, we may have missed some apps that would have met inclusion criteria for the review. Although we searched the largest app stores, there may be others that have additional relevant apps. In China, for example, Google Play is not available and other Android stores are used, which we could not search due to our geographic location and language barriers. Second, we conducted the search at one point in time (and repeated it several months later), but there may be new apps that have been released since our last search that we did not include in this review, and some reviewed apps may no longer be available or may have been updated. Another limitation is that we used the MARS scale, which does not consider end users’ evaluations of the apps or measure the extent to which users and reviewers agree in their assessment of apps. We did add a “characteristics” score to the original MARS scale which takes into account user ratings and number of downloads as listed on the app stores, but this was the only way we could capture users’ assessments of the apps. Finally, we did not examine the content of anti-abortion apps in depth and did not rate them using the MARS app, as our objective was to review apps that aided persons in obtaining or learning about abortion. In the future, examining the content of those apps in detail would be valuable.

This study also has several strengths. First, we evaluated the apps using a validated scale specifically developed for health-related mobile apps. Also, we had a multi-lingual team, which thoroughly reviewed apps in English, Spanish, and other languages. Two of the researchers are clinical abortion experts and used their expertise to develop a scale to measure the amount of abortion-specific information provided in an app. One of the researchers has worked in collaboration with designers and end-users to develop an abortion-related app and therefore, has special expertise in this field (of note, this app had not been released at the time of our search and was not included in the review).

In conclusion, this review provides an assessment of existing mobile apps that provide information about abortion. It is clear that few accurate, unbiased, functional, and appealing apps exist and that more could be developed to target users in different geographic areas. Reproductive health organizations and medical professionals should consider promoting high-quality apps to ensure that consumers access accurate information. Ultimately, though, we do not know, on a global scale, the extent to which persons who are seeking information about abortion are interested obtaining this information from apps as opposed to other digital media sources. The response to this question may vary based on age, geographic context, and Internet access. In our work to develop an abortion app in Venezuela, we engaged stakeholders to ensure we were designing a digital solution that was responsive to their needs. For most of the apps we reviewed, we were unable to determine whether stakeholders had influenced the design process. In the future, researchers should explore what individuals consider to be ideal digital sources of information about abortion, and app developers should consider user-centered design strategies to maximize their apps’ impact [19].

## Supporting information

S1 PRISMA Checklist PRISMA Checklist. (DOCX)
